# The Attitude of Parents Toward Their Children Receiving the COVID-19 Vaccine

**DOI:** 10.3390/children9091308

**Published:** 2022-08-28

**Authors:** Salmah Alghamdi

**Affiliations:** Maternity and Child Health Department, Faculty of Nursing, King Abdulaziz University, Jeddah 21589, Saudi Arabia; saalghamdi6@kau.edu.sa

**Keywords:** attitude, COVID-19, vaccinations, vaccine hesitancy, immunization, influenza vaccine

## Abstract

Parental attitudes towards childhood vaccination programs are important for successful delivery. Children were affected by COVID-19; however, parental attitudes towards childhood COVID-19 vaccination have not been fully assessed. The purpose is to assess parental hesitancy and attitudes about their children receiving a COVID-19 vaccination. This was a cross-sectional study using an electronically distributed questionnaire including a convenience sample of 123 Saudi Arabian parents of school-aged children between five and eleven years old. Most of the participants were mothers (77.2%) aged 31–40 years old (61%). The mean score of the questionnaire about parents’ attitudes towards the COVID-19 vaccine was M = 18.95, SD = 5.52. Only 39% of the parents were hesitant to have their children receive the seasonal influenza vaccine compared to 74% who were hesitant to have their children receive the COVID-19 vaccine. Most of the children (80%) did not receive the COVID-19 vaccine because of parental concerns about the vaccine’s side effects (49.5 %). Parents whose children received the COVID-19 vaccine (Median = 24, IQR = 9) had higher attitude scores than those whose children did not receive the vaccine (Median = 17, IQR = 6). The findings shed light on parental attitudes towards their children receiving a COVID-19 vaccination. Healthcare providers can build upon this study to improve parental attitudes towards childhood vaccination.

## 1. Introduction

Since the World Health Organization’s (WHO) global pandemic declaration for the coronavirus known as SARS-CoV-2 (COVID-19) in March 2020 [1], there has been an urgent need to prevent COVID-19 outbreaks. Globally, there have been more than five million deaths since the beginning of the COVID-19 pandemic [2]. COVID-19 has affected people from different countries and age groups, leading to mortality, morbidity, and economic changes.

The American Academy of Pediatrics reported over 11 million cases of COVID-19 among children, with a dramatic increase in 2022 caused by the Omicron variant [3]. Vaccination against COVID-19 is crucial to prevent the spread of the disease, and several vaccinations against COVID-19 have been approved and are available to adults aged 16 years and older. It is important that people accept vaccination programs for successful delivery to occur.

Vaccination against COVID-19 has recently become available for children; however, there is a need for more trials to confirm its efficacy in children [4]. Children can be asymptomatic while having confirmed COVID-19 positive results [5]. Although the COVID-19 morbidity and mortality rates among children are lower than among adults, children play a role in the community-based transmission of COVID-19 [5].

In clinical trials, researchers have assessed the efficacy of childhood vaccinations against COVID-19. Vaccinations allow children to safely engage in school and social activities. The Centers for Disease Control and Prevention [6] recommend that children aged five years and older be vaccinated against COVID-19.

Recently, the Ministry of Health (MOH) in the Kingdom of Saudi Arabia (KSA) allowed the COVID-19 vaccine to be administered to children five years and older [7]. The Pfizer–BioNTech vaccine was approved and available for children five years and older, and the Moderna vaccine is available for children 12 years and older [7].

Some parents are anxious about the vaccine’s safety and possible harm, which may increase their vaccine hesitancy [8]. The purposes of this study were to assess parents’ attitudes towards their children receiving a COVID-19 vaccination, parental hesitancy towards COVID-19 vaccination compared with the seasonal influenza vaccination, and the factors associated with parental attitudes towards childhood vaccination against COVID-19.

## 2. Materials and Methods

### 2.1. Study Design and Setting 

A quantitative cross-sectional research design was used. The sample was recruited from the community in the KSA through social media by using an online survey.

### 2.2. Sampling and Sample Size 

A convenience sample including parents of school-aged children between five and eleven years old was included. Parents of severely ill children or children with cancer, immunodeficiencies, or previous allergies to vaccination were excluded, because these factors may influence their candidacy to receive the vaccine. G*Power was used to run the power analysis for the purpose of sample size estimation [9]. A priori analysis was used with the following input parameters: alpha 0.05, power 0.95, and medium effect size 0.3. The suggested minimum sample size was 111.

### 2.3. Instrumentation 

A questionnaire contains of the following three sections was designed: (1) demographics including age, sex, and education level of parents and the number of siblings, (2) questions about the parents’ attitudes towards the COVID-19 vaccine; and (3) vaccine hesitancy. The items about attitudes towards the COVID-19 vaccine were developed from the Parent Attitudes about Childhood Vaccines (PACV) questionnaire [10] and by reviewing the relevant literature [5,10,11].

The attitude questionnaire assessed the beliefs and concerns about and trust in vaccine safety and efficacy. The adopted items from the PACV included eight items represented on five Likert-type scales. Calculation of the total attitude score was based on the eight items of the PACV questionnaire. The scoring for the answers was from one to five, taking into consideration the negatively worded question. The least favourable attitude was given a score of one, and the most favourable was given a score of five. Thus, a higher total score indicated a more positive attitude. Permission to use the questionnaire was obtained from the main author. The English version of the questionnaire was translated from the original English language into Arabic. The translated Arabic version was given to four Arabic experts to ensure the face validity and the clarity of meanings.

### 2.4. Data Collection Procedure 

An electronic version of the questionnaire was formed using Google Forms. Then, an electronic link for the questionnaire was sent and distributed through social media, including WhatsApp. The enrolment period was from February 2022 to April 2022.

### 2.5. Data Analysis 

Data were analysed using IBM® SPSS statistics 28 (Armonk, N.Y., USA). Descriptive statistics were utilized to describe the study variables including the mean, median, and frequencies. The comparison of the total score across variables with two groups was calculated using the independent samples t-test or the Mann–Whitney test. The Kruskal–Wallis test was used to compare the total scores across variables that had more than two groups. Multiple linear regression was conducted to study the factors associated with the outcome. The level of statistical significance was determined to be 0.05.

#### Ethical Considerations

Ethical approval was obtained from the Faculty of Nursing at King Abdulaziz University Jeddah, KSA. The serial number of the ethical approval is (2F. 04). All participation was completely voluntary and anonymous. Participants were assured that their information would be confidential and used for only research purposes. Interested participants read and approved the informed consent form before starting the electronic survey.

## 3. Results

### 3.1. Demographic Characteristics 

A total of 123 participants were included in the study. As shown in Table 1, of the parents who answered the questionnaire, 77.2 % were mothers. Most participants (61%) were 31–40 years old. More than one half of the children (56.9%) were five to eight years old, and 57.7% were female. Regarding employment, 48% of the participants worked full time (at least 35 h per week), and the highest percentage of them lived in the western regions (61%). For monthly household income, 35% of the respondents had more than SAR 13,000, and 29.3% of them earned between SAR 8001 and SAR 13,000. Most of the parents (46.3%) had a bachelor’s degree. More than half of the respondents had more than one sibling. More than one-half of the parents (56.1%) had had COVID-19, while only 37.4% of the children had had COVID-19.

### 3.2. Hesitancy Towards the COVID-19 Vaccination

Table 2 represents the hesitancy towards the COVID-19 vaccination. Only 39% of the respondents were hesitant for their children to receive the seasonal influenza vaccine compared with 74% who were hesitant for their children to receive the COVID-19 vaccine. More than one-half of the respondents (58.5%) had their children receive the COVID-19 vaccine so they could access children centres, such as daycare or school. Most of the children (80%) who did not receive the COVID-19 vaccine did not primarily because of concerns about side effects (49.5%).

### 3.3. Parents’ Attitudes Towards the COVID-19 Vaccine 

The mean score of the questionnaire on parents’ attitudes towards the COVID-19 vaccine was M = 18.95, SD = 5.52. The minimum score was 8, and the maximum was 39. A Kruskal–Wallis test was performed that compared the hesitancy for the child to receive a COVID-19 vaccination and the total attitude score. There was a statistically significant association between a parent’s hesitancy for the child to receive the COVID-19 vaccine and the total score of the parent’s attitude. Parents who were hesitant (Median = 16, IQR = 5) had lower attitude scores than those who were not hesitant (Median = 23.5, IQR = 8.5) *p*-value = 0.001. There was a statistically significant association between the child’s vaccination status and the total parent’s attitude score. The parents of children who received the COVID-19 vaccine (Median = 24, IQR = 9) had higher attitude scores than those whose children did not receive the vaccine (Median = 17, IQR = 6), *p*-value = < 0.001.

#### Factors Associated with Parental Attitudes

The total score of the attitude questionnaire compared to demographic variables was analysed. As shown in Table 3, a multiple linear regression was conducted, which reported the coefficients with 95% confidence intervals. Based on the results of the linear regression, child age was the factor associated with higher attitude scores towards vaccination. Parents with children in the age group nine to eleven years had a higher attitude score by an average of 2.59, (95% confidence interval, 0.35; 4.82) as compared with parents of children who were five to eight years old, *p*-value = 0.024.

## 4. Discussion

Vaccination against COVID-19 is the key to ending the transmission of the disease. Parental attitudes towards COVID-19 vaccination play a major role in their children’s immunization status. The purpose of this study was to assess parents’ attitudes towards their children receiving a COVID-19 vaccination, parental hesitancy towards COVID-19 vaccination in comparison to seasonal influenza vaccination, and the factors associated with parental attitudes towards childhood vaccination against COVID-19.

Most of the parents in this study (74%) were hesitant for their children to receive the COVID-19 vaccine compared with only 39% who were hesitant for their children to receive the seasonal influenza vaccine. A recent meta-analysis revealed that the intention toward influenza vaccination is related to the historical perception of the disease severity and the safety of the vaccine [12]. This implied that parental attitudes towards their children receiving a COVID-19 vaccine were different from their attitudes toward other vaccinations. The variation in the parental attitudes between COVID-19 and the seasonal influenza vaccines can be explained by the lack of information about the COVID-19 vaccination for children, as it has been recently approved for the use in children five years and older. Healthcare providers and stakeholders should make efforts to provide up-to-date information about vaccinating children against COVID-19.

The parents’ main reasons for not letting their children receive the COVID-19 vaccine according to the current study findings were primarily related to concerns about the safety of the vaccine (side effects), lack of information, and the perception that the COVID-19 disease does not greatly affect children. Consistently, the most reported reason in the literature for hesitancy about children receiving a COVID-19 vaccine was safety concerns [11,13,14]. Therefore, it is recommended that healthcare workers emphasize the evidence about the COVID-19 vaccine’s safety and efficacy for parents and children.

Additionally, there was a statistically significant association between the hesitancy for a child to receive the COVID-19 vaccine and the total score of the parental attitude questionnaire. This indicates that those who had a low attitude score towards having their children vaccinated against COVID-19 were less likely to have their children vaccinated against COVID-19. Consistently, a recent cross-sectional study from KSA reported that vaccine hesitancy was an important predictor of mothers’ intentions to have their children vaccinated against COVID-19 [11]. Likewise, it was reported that the attitudes regarding the COVID-19 vaccine were the main determinants of vaccination intention [15]. Thus, it is important for stakeholders to empower parents with clear information that enhances their attitudes towards having their children receive a COVID-19 vaccination.

The attitudes of parents towards having their children vaccinated against COVID-19 varied according to different personal and social factors. In the current study, the ages of children were found to be significantly associated with the parents’ attitudes. The parents with older children had a higher attitude score than those with younger children. A similar finding was found in a study from Qatar that revealed more hesitation among parents with younger adolescents than those with older adolescents [16].

The variation in the parental hesitancy about the COVID-19 vaccine may be related to the dissemination of the information and experience about vaccine effectiveness and safety for children because the information gradually progressed over time, moving from older groups to younger ones. Furthermore, findings from a large school survey in England and another in Germany revealed that older children were more likely to opt in to receiving vaccinations than younger children [17,18]. Because the COVID-19 vaccine has been approved and is now available for children five years or older, the awareness and reinforcement of the information about young children’s vaccination is needed. Moreover, there is a need for tailored interventions and communication strategies to target parents with younger children to increase their awareness of COVID-19 vaccination.

The study was a cross-sectional study; therefore, no causal relationships between parental attitude scores and other related factors can be inferred. Although the sample in the current study represented different geographical regions across the KSA, the small sample size may limit the generalizability of the findings.

## 5. Conclusions

The results of this study highlighted parental attitudes about having their children vaccinated against COVID-19. The findings revealed a statistically significant association between the child’s vaccination status and the total parent’s attitude score. Parental attitudes regarding COVID-19 vaccination can play a major role in improving the vaccination rates among children.

The findings implied that parental attitudes towards having their children vaccinated against COVID-19 were different from their attitudes towards influenza vaccinations. Because the main reason for not letting their children receive the COVID-19 vaccine was a concern about side effects, there is a critical need for increased awareness about the safety of the COVID-19 vaccine for children. These findings can be of great use for future researchers to build on in designing tailored awareness programs. Moreover, healthcare providers may use this study’s findings to predict parental attitudes and to improve parental vaccination awareness.

## Figures and Tables

**Table 1:** Demographic characteristics.

	*n*	%
Parents who participated		
Mother	95	77.2
Father	28	22.8
Parent’s age		
Between 20 and 30	13	10.6
Between 31 and 40	75	61.0
41 or above	35	28.5
Child’s age		
Between 5 and 8 years	70	56.9
Between 9 and 11 years	53	43.1
Sex of Child		
Male	52	42.3
Female	71	57.7
Parent’s Employment Status		
Working full-time (at least 35 h per week)	59	48.0
Working part-time	19	15.4
Not working	45	36.6
Location		
North region	10	8.1
West region	75	61.0
South region	14	11.4
East region	13	10.6
Middle region	11	8.9
Household’s monthly Income		
SAR 8000 or less	24	19.5
SAR 8001–SAR 13000	36	29.3
More than SAR 13000	43	35.0
Don’t know	20	16.3
Parent’s educational level		
Below high school	6	4.9
High school	26	21.1
Bachelor’s degree	57	46.3
Master’s degree or higher	34	27.6
Number of siblings		
None	8	6.5
One sibling	21	17.1
More than one sibling	94	76.4
The parent had COVID-19 previously		
No	47	38.2
Yes	69	56.1
Don’t know	7	5.7
The child had COVID-19 previously		
No	68	55.3
Yes	46	37.4
Don’t know	9	7.3

**Table 2:** Hesitancy toward COVID-19 vaccination.

	*n*	%
I am hesitant about the childhood seasonal influenza vaccination		
No	64	52.0
Yes	48	39.0
Don’t know	11	8.9
I am hesitant about the childhood COVID-19 vaccine		
No	28	22.8
Yes	91	74.0
Don’t know	4	3.3
In order to enter daycare or school, I had my child receive a vaccine		
No	51	41.5
Yes	72	58.5
Did your child receive the COVID-19 vaccine?		
No	99	80.5
Yes	24	19.5
Reasons why your child did not receive the COVID-19 vaccine		
Concerns about the side effects of the vaccine	49	49.5
Lack of information about the vaccine for children	15	15
Children’s immunity is good	8	16.3
COVID-19 disease does not greatly affect children	13	13
Others	14	14

**Table 3:** Multiple linear regressions for the factors associated with parents’ attitude towards vaccination. Bold means significance.

	Coefficient	*p*-Value	95% CI of the coefficient	
Parents who filled out the questionnaire				
Mother	Ref			
Father	2.60	0.087	−0.38	5.58
Parent’s age				
Between 20 and 30	Ref			
Between 31 and 40	−0.23	0.905	−4.08	3.62
41 or above	1.95	0.358	−2.24	6.13
Child’s age				
Between 5 and 8 years	Ref			
Between 9 and 11 years	2.59	**0.024**	0.35	4.82
Sex of Child				
Male	Ref			
Female	−0.81	0.469	−3.03	1.40
Parent’s employment Status				
Working full-time (at least 35 h per week)	Ref			
Working part-time	−0.77	0.692	−4.61	3.07
Not working	2.07	0.157	−0.81	4.95
Location				
North regions	Ref			
West regions	1.09	0.620	−3.26	5.44
South regions	1.97	0.457	−3.26	7.20
East regions	2.41	0.402	−3.27	8.10
Middle regions	4.56	0.113	−1.10	10.22
Household’s monthly Income in Saudi Arabian Riyal (SAR)				
SAR 8000 or less	Ref			
SAR 8001–SAR 13000	−1.14	0.485	−4.37	2.09
More than SAR 13000	−1.10	0.522	−4.51	2.30
Don’t know	−1.60	0.419	−5.52	2.31
Parent’s Educational Level				
Below high school	Ref			
High school	−0.94	0.733	−6.38	4.50
Bachelor’s degree	−1.13	0.667	−6.34	4.08
Master’s degree or higher	0.02	0.995	−5.64	5.68
Number of siblings				
None	Ref			
One sibling	−0.50	0.841	−5.47	4.46
More than one sibling	−0.20	0.932	−4.84	4.45
The parent had COVID-19 disease				
No	Ref			
Yes	−0.95	0.480	−3.59	1.70
Don’t know	1.56	0.613	−4.55	7.67
The child had COVID-19 disease				
No	Ref			
Yes	−0.02	0.991	−2.77	2.74
Don’t know	−1.76	0.515	−7.09	3.58

## Data Availability

The data are available from the author on request.
